# Impact of Pre-Revascularization and Post-Revascularization Cardiac Arrest on Survival Prognosis in Patients With Acute Myocardial Infarction and Following Emergency Percutaneous Coronary Intervention

**DOI:** 10.3389/fcvm.2021.705504

**Published:** 2021-11-19

**Authors:** Changzuan Zhou, Qingcheng Lin, Guangze Xiang, Mengmeng Chen, Mengxing Cai, Qianli Zhu, Rui Zhou, Weijian Huang, Peiren Shan

**Affiliations:** ^1^Department of Cardiology, The Key Laboratory of Cardiovascular Disease of Wenzhou, The First Affiliated Hospital of Wenzhou Medical University, Wenzhou, China; ^2^Department of Cardiology, Wenzhou Hospital of Integrated Traditional Chinese and Western Medicine, Wenzhou, China; ^3^Department of Cardiology, Wenzhou People's Hospital, Wenzhou, China

**Keywords:** cardiac arrest, resuscitation, acute myocardial infarction, survival, percutaneous coronary intervention (PCI)

## Abstract

**Objectives:** To evaluate the effects of occurrence and timing of sudden cardiac arrest (SCA) on survival in patients with acute myocardial infarction (AMI) who underwent emergency percutaneous coronary intervention (PCI).

**Methods:** We analyzed 1,956 consecutive patients with AMI with emergency PCI from 2014 to 2018. Patients with cardiac arrest events were identified, and their medical records were reviewed.

**Results:** Patients were divided into non-cardiac arrest group (NCA group, *n* = 1,724), pre-revascularization cardiac arrest (PRCA group, *n* = 175), and post-revascularization SCA (POCA group, *n* = 57) according to SCA timing. Compared to NCA group, PRCA group and POCA group presented with higher brain natriuretic polypeptide (BNP), more often Killip class 3/4, atrial fibrillation, and less often completed recovery of coronary artery perfusion (all *p* < 0.05). Both patients with PRCA and POCA showed increased 30-day all-cause mortality when compared to patients with NCA (8.0 and 70.2% vs. 2.9%, both *p* < 0.001). However, when compared to patients with NCA, patients with PRCA did not lead to higher mortality during long-term follow-up (median time 917 days) (16.3 vs. 18.6%, *p* = 0.441), whereas patients with POCA were associated with increased all-cause mortality (36.3 vs. 18.6%, *p* < 0.001). Multivariate analysis identified Killip class 3/4, atrial fibrillation, high maximum MB isoenzyme of creatine kianse, and high creatinine as predictive factors for POCA. In Cox regression analysis, POCA was found as a strong mortality-increase predictor (HR, 8.87; 95% CI, 2.26–34.72; *p* = 0.002) for long-term all-cause death.

**Conclusions:** POCA appeared to be a strong life-threatening factor for 30-day and long-term all-cause mortality among patients with AMI who admitted alive and underwent emergency PCI. However, PRCA experience did not lead to a poorer long-term survival in patients with AMI surviving the first 30 days.

## Introduction

Sudden cardiac arrest (SCA) was one of the leading causes of mortality in patients with coronary artery disease (CAD). Among survivors of cardiac arrest occurring pre-hospitalization, CAD was present in more than 70% in one study (1), with total occlusion of culprit vessels in half of them; and 71% in another study, with 43% presenting with acute myocardial infarction (AMI) (2). The coronary angiographic assessment documented culprit vessels in 92.7% of SCA survivors presenting with ST-elevation myocardial infarction (STEMI) and in 69.2% of those without ST-segment elevation. Coronary angiography and percutaneous coronary intervention (PCI) are key components of the management of SCA complicated with STEMI or non-STEMI (NSTEMI) (3, 4). Despite significant advances in life-supporting and post-arrest management, high in-hospital mortality and poor post-hospitalization prognosis were still observed in SCA populations. Recent studies have also yielded inconsistent results on whether emergency PCI benefits survival in patients with SCAs (5–7). Moreover, a long-term outcome analysis performed in non-selected patients with AMI, regarding influence of timing of SCA, is lacking. Here, we investigated the impact of occurrence and timing (pre-revascularization and post-revascularization) of witnessed SCA on short-term and long-term mortality in non-selected patients who underwent emergency PCI.

## Methods

### Study Setting and Study Population

This study enrolled patients with AMI who underwent emergency coronary angiography and subsequent PCI between January 2014 and October 2018 in the First Affiliated Hospital of Wenzhou Medical University. All patients were initially diagnosed with AMI at admission and underwent emergency PCI. This research was approved by the Ethics Committee of the First Affiliated Hospital of Wenzhou Medical University and performed according to the principles of the Declaration of Helsinki. Participants provided written informed consent, and their details were kept anonymized. The diagnosis of AMI was based on the detection of the rise of troponin I above 99th percentile of upper reference limit together with at least one of the following evidence of myocardial infarction: (1) symptoms of ischemia, including various combinations of chest discomfort, (2) ECG changes, indicative of new ischemia, (3) development of pathological Q waves in the ECG, and (4) imaging evidence of new loss of viable myocardium or new regional wall motion abnormality. Patients with AMI with SCA were classified as pre-revascularization cardiac arrest (PRCA) group and post-revascularization cardiac arrest (POCA) group according to the timing of SCA. Patients with AMI without SCA were enrolled in non-cardiac arrest (NCA) group. The exclusion criteria were: (1) incomplete invasive coronary angiography image data; (2) missing records on SCA occurrence, and (3) clinical and angiography data not supporting a diagnosis of AMI.

### Data Collection

To ensure data integrity, consistency, and comparability, the investigators were trained before inputting the required information extracted from the clinical records of each patient. The demographic and medical history data included age, sex, hypertension, diabetes mellitus, hyperlipidemia, cerebrovascular disease history, previous smoking history, and alcohol drinking history. The blood laboratory examinations, including brain natriuretic polypeptide (BNP), D–D dimer, lactate, creatinine, glucose, triglyceride, and low-density lipoprotein cholesterol (LDL-c) were determined upon hospital admission. Clinical presentation data included timing of SCA (PRCA and POCA), type of AMI (e.g., STEMI and NSTEMI), atrial fibrillation, use of vasoactive drugs (including inotropes, dobutamine, vasopressors, etc.), and use of intra-aortic balloon pump (IABP). Invasive data collected from cardiac catheterization laboratory records included percentage stenosis of affected coronary vessel, culprit coronary vessel [e.g., left anterior descending artery (LAD), left main (LM) artery, left circumflex artery (LCX), and right coronary artery (RCA)], thrombolysis in myocardial infarction (TIMI) flow grade before and after intervention, implantation of drug-eluting stents, use of thrombus aspiration, and corrected TIMI frame count (CTFC). Culprit vessel calcification was qualitatively stratified in four degrees by reviewing coronary angiographic images. Calcification class 0, no calcification is visible in culprit vessels; calcification class 1, mild and obscure calcification, which can only be observed in culprit vessels when the ventricular walls were moving; calcification class 2, moderate and clear calcification, which can only be observed in culprit vessels when the ventricular walls were moving; and calcification class 4, remarkable calcification can be observed in culprit vessels whether the ventricular walls were moving or not.

### Definitions

Sudden cardiac arrest was defined as the absence of heartbeats and respiration requiring CPR for recovery. Shockable rhythm was defined as pulseless ventricular tachycardia (pVT) and ventricular fibrillation (VF), which could benefit from electric defibrillation, whereas non-shockable rhythm contained other pulseless electrical activities and asystole rhythm. LM or ostial LAD stenosis was defined as more than 50% vessel stenosis in LM or ostial LAD. Three-vessel disease was defined as more than three vessels stenosis >50%. CTFC was calculated from the first frame in which dye fully entered the artery to the time point contrast fluid reached standardized distal landmarks at a frame rate of 30 frames/s during coronary angiography. The developed symptom of heart failure included New York Heart Association class III/IV dyspnea, orthopnea, and rales greater than one-third lung fields thought to be related to cardiac dysfunction. Ventricular arrhythmia was defined as sustained pVT and VF.

The main outcomes of interest were 30-day all-cause mortality and long-term survival prognosis in 30-day survivors. Major adverse cardiac and cerebrovascular events (MACCE) were defined as a composite of recurrent myocardial infarction, admission for unstable angina, ventricular tachycardia/ventricular fibrillation, cerebral ischemic, and major bleeding event.

### Follow-Up and Survival Data

The death dates were obtained by checking inpatient department records and telephone follow-up. The last telephone follow-up was 1 June, 2019. And patients who lost to telephone follow-up were noted as censored at the last date they were known to be alive. The days from admission until censoring or death were calculated to be the follow-up time.

### Statistical Analysis

Normally distributed continuous variables were analyzed by the Student–Newman–Keuls (SNK) test and presented as mean ± SD. The Wilcoxon rank-sum test was used to compare medians of non-normally distributed continuous variables that were analyzed by the Wilcoxon rank-sum test and presented as medians (25, 75th percentile). Differences between categorical variables were assessed with the Chi-square test, respectively. The univariate and multivariate stepwise Logistic regression analyses were used to identify POCA-related factors. Landmark analysis was performed to evaluate 30-day and long-term survival prognosis, and the Log-rank test was used to compare Kaplan–Meier event rates among groups. The Kaplan–Meier curves were plotted by MedCalc 18 (MedCalc Software, Ostend, Belgium). Cox proportional hazards regression was used to determine risk factors associated with mortality during follow-up period in the studied population. A two-tailed *p* < 0.05 was considered statistically significant. All statistical analyses were performed using STATA 13 (StataCorp LP, College Station, TX, USA).

## Results

### Baseline Clinical Characteristics

**Figure 1:**
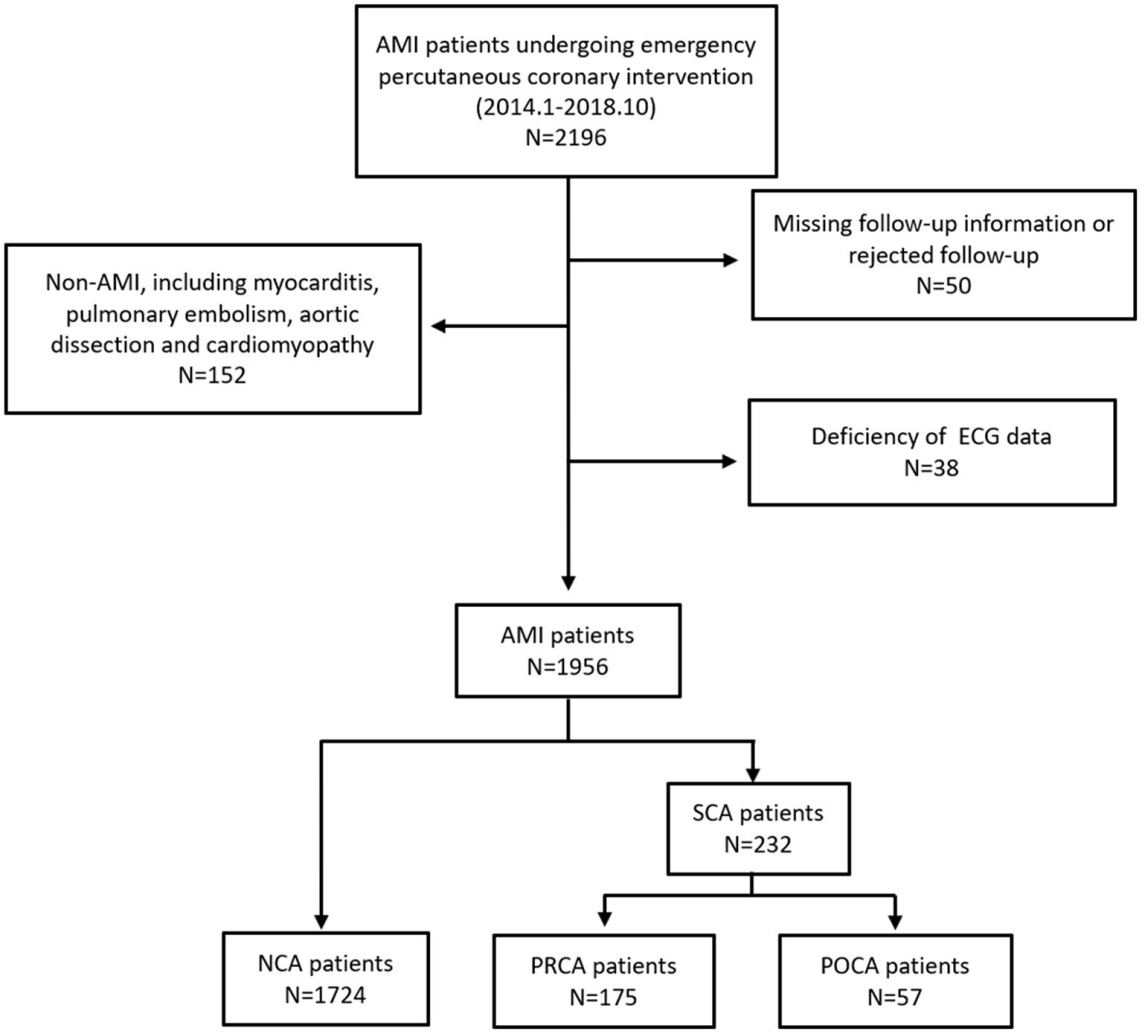
Study population. NCA, non-cardiac arrest; POCA, post-revascularization cardiac arrest; PRCA, pre-revascularization cardiac arrest.

This study initially enrolled 2,154 patients, and the final dataset consisted of 1,956 patients with a discharge diagnosis of AMI, including STEMI and NSTEMI during the study period, as described in Figure 1. Of those, SCA was recorded in 232 resuscitated patients, with 175 as PRCA and 57 as POCA. We found 5 patients experienced both PRCA and subsequent POCA events. These 5 patients were enrolled in PRCA group. As shown in Table 1, some baseline clinical characteristics of patients, including alcohol use, hypertension, and hyperlipidemia, were evenly distributed among the three groups. The NCA group and POCA group showed similar male gender proportion, 60.2 and 56.1%, respectively (*p* = 0.540), whereas in the PRCA group the male gender (72.0%) percentage was significantly higher than that in the NCA group (*p* = 0.002). Compared to the NCA group, the POCA group was more likely to be older age, more diabetes, lower proportion of smokers, and poorer ejection fraction (all *p* < 0.05). Compared to the NCA group, both PRCA and POCA groups showed higher levels of BNP, D–D dimer, maximum MB isoenzyme of creatine kianse (CK-MB), lactate, and creatinine (all *p* < 0.05). In the POCA group, the BNP was nearly three times the level of the NCA group (287.5 vs. 119.0 pg/ml, *p* < 0.001), and serum lactate was more than twice of the NCA group (6.0 vs. 2.2 mmol/L, *p* < 0.001). We also found higher D–D dimer concentration in patients with PRCA and POCA than in patients with NCA (*p* < 0.001).

**Table 1:** Baseline characteristics.

	**NCA**	**PRCA**	**POCA**	***p*-value^**1**^**	***p*-value^**2**^**
	***N* = 1724**	***N* = 175**	***N* = 57**		
Demographics					
Male gender	1,037 (60.2)	126 (72.0)	32 (56.1)	0.002	0.540
Age (years)	63.6 ± 12.9	61.5 ± 13.3	68.4 ± 13.1	0.053	0.001
Smoking (current or past)	886 (51.5)	89 (50.9)	18 (31.6)	0.863	0.003
Alcohol (current or past)	395 (23.0)	36 (20.6)	11 (19.3)	0.469	0.515
Past medical history					
Hypertension	934 (54.4)	102 (58.3)	33 (57.9)	0.321	0.599
Diabetes	361 (21.0)	30 (17.1)	20 (35.1)	0.230	0.011
Hyperlipidemia	446 (25.9)	50 (28.5)	15 (26.3)	0.846	0.303
Stroke	54 (3.1)	7 (4.0)	6 (10.5)	0.541	0.011
Admission labs					
BNP (pg/ml)	119.0 (47.0–328.0)	168.0 (54.5–520.0)	287.5 (74.5–1926.0)	0.009	<0.001
D–D dimer (mg/L)	1.0 (0.7–1.4)	1.4 (0.8–3.8)	1.7 (1.0–3.7)	<0.001	<0.001
Maximum CK-MB(U/L)	251.0 (124.0–428.0)	269.0 (123.0–514.0)	542.0 (223.5–765.3)	0.027	0.002
Lactate (mmol/L)	2.2 (1.6–3.0)	2.5 (1.8–3.8)	6.0 (4.2–9.5)	<0.001	<0.001
Creatinine (μmol/L)	73.0 (62.0–88.0)	78.0 (67.0–98.0)	145.0 (76.5–189.5)	0.001	<0.001
Glucose (mmol/L)	7.6 ± 4.5	7.7 ± 3.9	9.3 ± 5.7	0.750	0.005
Triglyceride (mmol/L)	1.5 ± 1.4	1.4 ± 0.8	1.2 ± 0.7	0.306	0.046
LDL-c (mmol/L)	3.1 ± 1.0	3.0 ± 1.1	2.8 ± 1.1	0.279	0.044
Echocardiography					
LVEF (%)	49.1 ± 9.2	47.3 ± 8.8	42.4 ± 7.9	0.119	<0.001
LVEDD (mm)	50.1 ± 5.9	49.9 ± 6.0	50.6 ± 5.7	0.667	0.476
LA (mm)	39.5 ± 9.4	37.9 ± 6.0	39.9 ± 5.4	0.038	0.713
STEMI	1331 (77.2)	147 (84.0)	46 (80.7)	0.044	0.631
NSTEMI	393 (22.8)	28 (16.0)	11 (19.3)		
Admission SBP (mmHg)	118.5 ± 31.1	113.4 ± 21.7	110.9 ± 14.5	0.034	0.064
Admission DBP (mmHg)	71.4 ± 14.5	70.9 ± 14.4	66.6 ± 11.5	0.685	0.016
Admission Killip class					
1/2	1,590 (92.2)	122 (69.7)	13 (22.8)	<0.001	<0.001
3/4	134 (7.8)	53 (30.3)	44 (77.2)	<0.001	<0.001
Ventilator	66 (3.8)	43 (28.7)	40 (70.2)	<0.001	<0.001
Heart failure	189 (11.0)	27 (15.4)	22 (38.6)	0.076	<0.001
Vasoactive drugs use	82 (4.8)	34 (19.4)	20 (35.1)	<0.001	<0.001
IABP	52 (3.0)	13 (8.5)	19 (33.3)	0.001	<0.001
Electrophysiological dysfunction					
AVB	33 (1.9)	16 (9.1)	10 (17.5)	<0.001	<0.001
LBBB	24 (1.4)	14 (8.0)	9 (15.8)	<0.001	<0.001
Atrial fibrillation	110 (6.4)	19 (10.9)	18 (31.6)	0.025	<0.001
New-onset atrial fibrillation	36 (2.1)	9 (5.1)	11 (19.3)	0.031	<0.001
Atrial flutter	31 (1.8)	11 (6.3)	6 (10.5)	0.001	0.001
VF/pVT	22 (1.3)	35 (20.0)	21 (36.8)	<0.001	<0.001
*Discharge medication					
Aspirin	1,600 (94.7)	151 (92.1)	18 (100)	0.154	0.620
P2Y_12_ antagonist	1,595 (94.4)	155 (94.5)	15 (83.3)	0.967	0.131
Statins	1,620 (95.9)	153 (93.3)	18 (100)	0.115	0.474
β-receptor inhibitor	1,221 (72.3)	111 (67.7)	13 (72.2)	0.210	1.000
ACEI/ARB	1,072 (63.5)	93 (56.7)	8 (44.4)	0.087	0.096
Aldosterone receptor antagonist	411 (24.3)	43 (26.2)	3 (16.7)	0.593	0.587

Data are presented as mean ± SD, medians (25, 75th) or n (%). p-value^1^, NCA group vs. PRCA group; p-value^2^, NCA group versus POCA group. *Discharge medication were analyzed in discharge survivors, with 1,689 patients in the NCA group, 164 patients in the PRCA group and 18 patients in the POCA group. AVB, atrioventricular block; BNP, brain natriuretic peptide; CK-MB, MB isoenzyme of creatine kianse; LA, left atrial diameter; LBBB, left bundle branch block; LDL-c, low-density lipoprotein cholesterol; LVEF, left ventricular ejection fraction; LVEDD, left ventricular end-diastolic dimension; IABP, intra-aortic balloon pump; IHCA, in-hospital cardiac arrest; NCA, non-cardiac arrest; NSTEMI, non-ST-segment elevation myocardial infarction; PCI, percutaneous coronary intervention; PRCA, pre-revascularization cardiac arrest; POCA, post-revascularization cardiac arrest; SCA, sudden cardiac arrest; STEMI, ST-segment elevation myocardial infarction; DBP, dilation blood pressure; SBP, systolic blood pressure; VF, ventricular fibrillation; pVT, pulseless ventricular tachycardia.

Compared to patients in the NCA group, patients with PRCA were more likely to be STEMI (84.0 vs. 77.2%, *p* = 0.044), whereas there was no significant of clinical presentation between the NCA group and POCA group. Patients with SCA had more often admission Killip class 3/4, ventilator use, IABP, and vasoactive drugs use compared to patients in the NCA group (all *p* < 0.05). Moreover, patients with SCA had more frequent heart failure and electrophysiological dysfunction, including atrioventricular block, left bundle branch block, ventricular arrhythmia (VF/pVT), atrial fibrillation, and atrial flutter (all *p* < 0.05).

As presented in Table 2, no intergroup difference was observed in discharge medication, which included aspirin, P2Y12 antagonist, β-receptor inhibitor, angiotensin-converting enzyme inhibitor, angiotensin receptor blockers, statins, and aldosterone receptor antagonists (all *p* > 0.05).

**Table 2:** Procedural characteristics.

	**NCA**	**PRCA**	**POCA**	***p*-value^**1**^**	***p*-value^**2**^**
	***N* = 1,724**	***N* = 175**	***N* = 57**		
Culprit vessel					
LAD	887 (51.5)	72 (41.1)	25 (43.9)	0.009	0.259
LCX	225 (13.1)	12 (6.9)	8 (14.0)	0.018	0.828
LM	31 (1.8)	2 (1.1)	6 (10.5)	0.763	<0.001
RCA	578 (33.5)	87 (49.7)	16 (28.1)	<0.001	0.390
LM or ostial LAD stenosis	463 (26.9)	32 (18.3)	22 (38.6)	0.014	0.050
Vessel occlusion	1,094 (63.5)	114 (65.1)	36 (63.2)	0.659	0.963
Culprit vessel stenosis (%)	96.6 ± 7.9	96.8 ± 5.8	97.4 ± 5.1	0.678	0.117
Three-vessel disease	466 (27.0)	52 (29.7)	24 (42.1)	0.447	0.012
Calcification degree in culprit lesion					
0/1	1,528 (88.6)	160 (91.4)	48 (84.2)	0.262	0.304
2	110 (6.4)	10 (5.7)	3 (5.3)	0.730	0.935
3	86 (5.0)	5 (2.9)	6 (10.5)	0.676	0.120
CTO in non-culprit vessel	114 (6.6)	15 (2.9)	5 (8.8)	0.326	0.427
Stent implantation	1,496 (87.0)	150 (85.7)	43 (76.8)	0.624	0.026
Thrombus aspiration	651 (37.9)	84 (48.0)	24 (42.1)	0.009	0.521
ICD/CRTD	7 (0.4)	2 (1.1)	0	0.198	0.796
Pre-PCI TIMI					
0/1	1,309 (76.1)	135 (77.2)	45 (79.0)	0.925	0.752
2	166 (9.6)	13 (7.4)	2 (3.5)	0.343	0.164
3	246 (14.3)	27 (15.4)	10 (17.5)	0.677	0.488
Post-PCI TIMI					
0/1	22 (1.3)	4 (2.3)	2 (3.5)	0.079	0.094
2	92 (5.3)	16 (9.1)	7 (12.3)	0.007	0.009
3	1610 (93.4)	155 (85.5)	48 (86.0)	0.001	0.002
Post-PCI SBP (mmHg)	122.6 ± 22.7	115.1 ± 23.4	110.5 ± 19	<0.001	<0.001
Post-PCI DBP (mmHg)	74.6 ± 14.5	71.4 ± 14.1	66.9 ± 12.6	0.012	<0.001
Post-PCI CTFC	38.0 (30.0–50.0)	40.0 (32.0–58.0)	45.0 (35.5–59.5)	0.037	0.012

*Data are presented as mean ± SD, n (%) or medians (25, 75th). p-value^1^, NCA group vs. PRCA group; p-value^2^, NCA group vs. POCA group. AMI, acute myocardial infarction; CRTD, cardiac resynchronization therapy defibrillator; CTFC, corrected TIMI frame count; CTO, chronic total occlusion; ICD, implantable cardioverter defibrillator; LAD, left anterior descending; LCX, left circumflex coronary artery; LM, left main artery; RCA, right coronary artery; DBP, dilation blood pressure; SBP, systolic blood pressure; TIMI, thrombolysis in myocardial infarction; PCI, percutaneous coronary intervention; NCA, non-cardiac arrest; PRCA, pre-revascularization cardiac arrest; POCA, post-revascularization cardiac arrest*.

### Procedural Characteristics

According to cardiac catheterization laboratory data (Table 2), the culprit vessel of LM artery was more likely to be observed in the POCA group when compared to that in the NCA group (10.5 vs. 1.8%, *p* < 0.001). There was a higher proportion of LM or ostial LAD stenosis in patients with POCA than in patients with NCA (38.6 vs. 26.9%, *p* = 0.050). Culprit vessel of LAD and LCX were less commonly observed in the PRCA group when compared to the NCA group (*p* = 0.009 and *p* = 0.018). However, the average stenosis percentage of culprit vessel in the PRCA group and POCA group was similar to that in the NCA group (all *p* > 0.05).

The pre-PCI TIMI flow degree in culprit vessel in PRCA and POCA groups was not significantly different compared to that in the NCA group; however, a lower percentage of TIMI flow three restorations was observed in the PRCA group and POCA group compared to that in the NCA group (PRCA and POCA vs. NCA; 85.5% and 86.0% vs. 93.4%, *p* = 0.001 and *p* = 0.002, respectively). We found a higher proportion of three-vessel disease in the POCA group compared to that in the NCA group (42.1% vs. 27.0%, *p* = 0.012). However, we did not observe the significant intergroup difference in culprit vessel calcification, chronic total occlusion (CTO), and collateral circulation between patients without and with SCA regardless of type (all *p* > 0.05).

In addition, post-PCI systolic and diastolic blood pressures were significant lower in patients with POCA and PRCA compared to those in patients with NCA (all *p* < 0.05). Significant higher CTFC was observed in patients with SCA (PRCA and POCA) than in patients without SCA (40.0 and 45.0 vs. 38.0 frames, *p* = 0.037 and *p* = 0.012, respectively).

### 30-Day and Long-Term Survival Rate

Overall, 95.7% (1,871/1,956) of the patients were alive at the time of discharge. The in-hospital mortality rates of PRCA and POCA groups were higher than that of the NCA group (6.3 and 68.4% vs. 2.0%; both *p* < 0.05) (Table 3). Because a significant portion of patients in Asian countries opted to return home when the living chance was very low and their status at discharge were alive, we chose 30-day mortality to evaluate short-term survival. The median follow-up time from day 30 to end of the study in 30-day survivors was 917 days. Additionally, the 1-year follow-up rate and 3-year follow-up rate resulted to be 83.2 and 45.1%, respectively.

**Table 3:** Cumulative mortality in patients with AMI stratified by timing of SCA.

	**NCA**	**PRCA**	**POCA**	***p*-value^**1**^**	***p*-value^**2**^**
	***N* = 1724**	***N* = 175**	***N* = 57**		
In-hospital mortality	35 (2.0)	11 (6.3)	39 (68.4)	0.002	<0.001
30-day mortality	50 (2.9)	14 (8.0)	40 (70.2)	<0.001	<0.001
^*^Long-term mortality rate (95%CI)	18.6 (12.2–27.8)	16.3 (8.7–23.9)	36.3 (7.7–64.7)	0.441	<0.001
^*^Long-term follow-up MACCE	51 (3.0)	11 (6.7)	1 (5.6)	0.020	0.429

*Results are given as n (%) or percentage with 95% CI. p-value^1^, NCA group vs. PRCA group; p-value^2^, NCA group vs. POCA group*.

* *Day 30 to end of study, median follow-up time: 917 days (25, 75^th^ percentile: 474, 1,326). AMI, acute myocardial infarction; SCA, sudden cardiac arrest; MACCE, major adverse cardiac and cerebrovascular events; NCA, non-cardiac arrest; PRCA, pre- revascularization cardiac arrest; POCA, post-revascularization cardiac arrest*.

**Figure 2:**
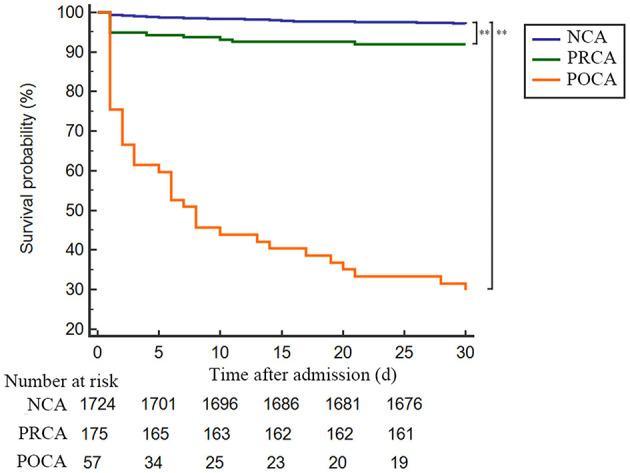
Kaplan–Meier plot of 30-day survival probability of AMI patients underwent emergency PCI stratified by timing of cardiac arrest. Admission to 30-day survival curves for NCA patients (blue line), PRCA patients (green line) and POCA patients (orange line). **p < 0.001. AMI, acute myocardial infarction; NCA, non-cardiac arrest; PCI, percutaneous coronary intervention; POCA, post-revascularization cardiac arrest; PRCA, pre- revascularization cardiac arrest.

**Figure 3:**
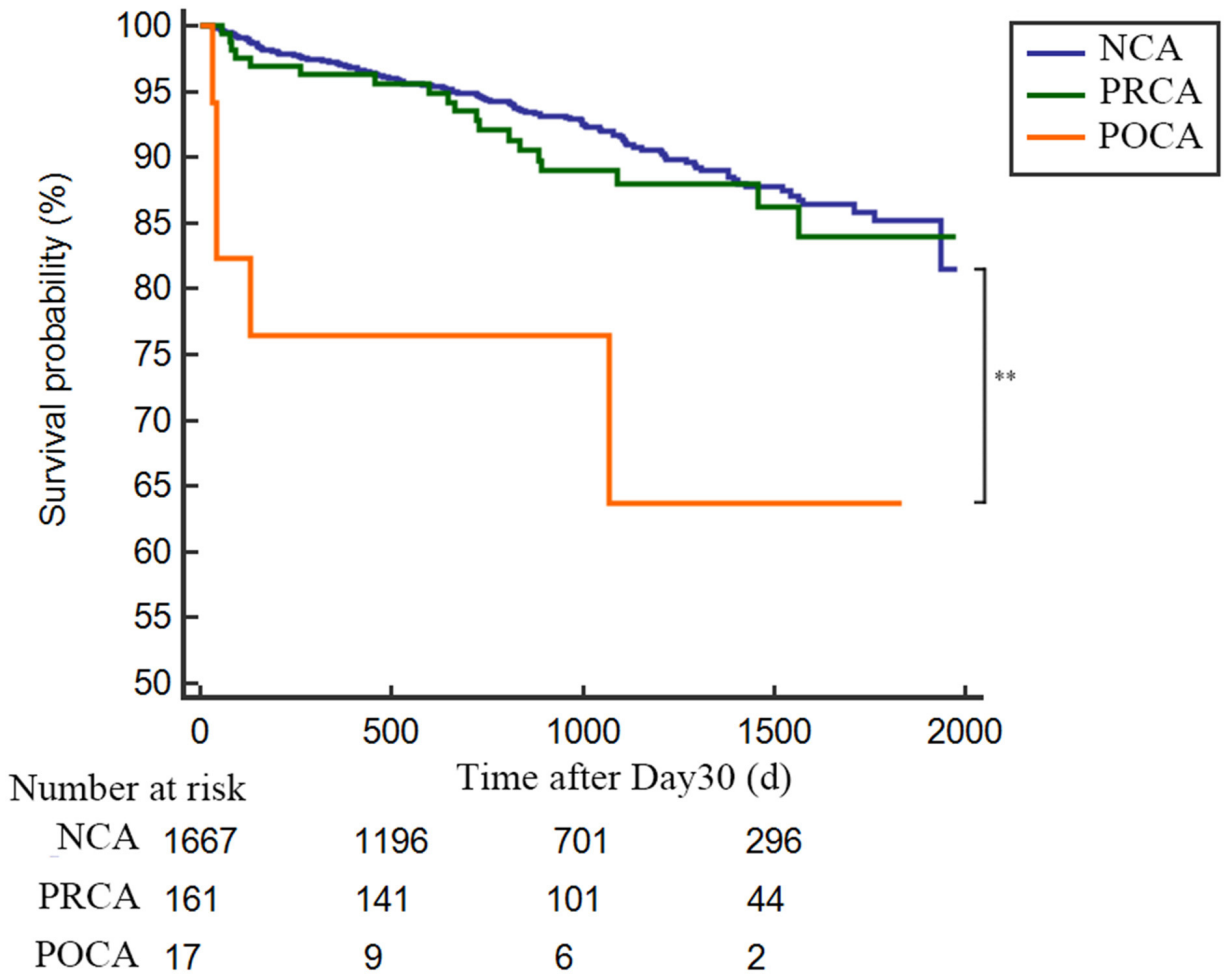
Landmark analysis for long-term survival rate in 30-day survivors in AMI patients either complicated or not with cardiac arrest. Day 30 to long-term survival curves for NCA patients (blue line), PRCA patients (green line) and POCA patients (orange line). **p < 0.001. NCA, non-cardiac arrest; POCA, post-revascularization cardiac arrest; PRCA, pre-revascularization cardiac arrest.

As demonstrated in Table 3 and Figure 2, the 30-day survival was lower both in patients with PRCA and POCA when compared to patients with NCA (8.0% and 70.2% vs. 2.9%, both *p* < 0.001). Among 30-day survivors, patients with POCA still showed worse survival prognosis during long-term follow-up than those without SCA occurrence (*p* < 0.001) (Table 3 and Figure 3). However, long-term cumulative mortality rate was not significantly different between NCA and PRCA groups among 30-day survivors (18.6 vs. 16.3%, *p* = 0.441).

**Figure 4:**
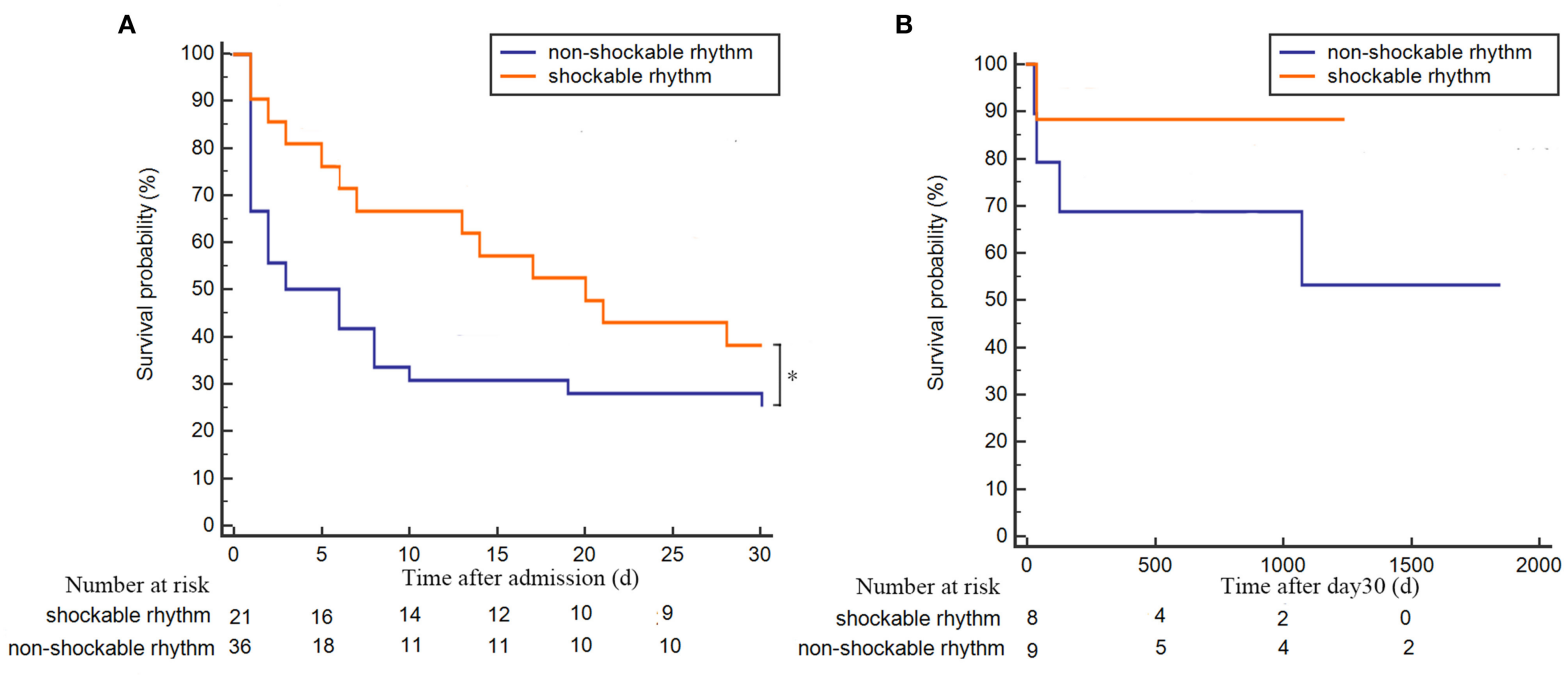
Kaplan–Meier plot of survival probability for POCA patients with shockable or non-shockable rhythm. **(A)** 30-day follow-up survival curve for POCA patients with shockable rhythm (orange line) or non-shockable rhythm (blue line); **(B)** long-term follow-up survival curve of POCA patients with shockable rhythm (orange line) or non-shockable rhythm (blue line) in 30-day survivors. *p < 0.05. POCA, post-revascularization cardiac arrest; PRCA, pre-revascularization cardiac arrest.

In the POCA group, we recorded shockable rhythm (VF/pVT) in 21 patients (36.8%) and non-shockable rhythm (pulseless electrical activity/asystole) in the other 36 patients (63.2%). Among POCA population, we found that non-shockable rhythm was associated with poor 30-day survival prognosis but similar survival prognosis in long-term follow-up, compared to shockable rhythm patients (*p* = 0.032 and *p* = 0.232, respectively) (Figure 4).

### Independent Predictors for POCA

In univariate Logistic binary regression analysis, age, Killip degree, diabetes, BNP, LVEF, Lactate, D–D dimer, creatinine, CK-MB, post-PCI SBP and DBP, atrial fibrillation, heart failure, culprit vessel of LM, and post-PCI CTFC were identified predictors for POCA occurrence. After adjustment for confounders, multivariate Logistic regression showed that Killip class 3/4 (OR 45.73, 95%CI 12.03–173.78, *p* < 0.001), high creatinine (OR 1.33, 95%CI 1.09–1.64, *p* = 0.006), atrial fibrillation (OR 5.39, 95%CI 1.45–20.04, *p* = 0.001), and higher maximum CK-MB (OR 1.30, 95%CI 1.09–1.54, *p* = 0.002) were independent risk factors for POCA (Table 4).

**Table 4:** Independent predictors of POCA occurrence.

**IHCA-associated factor**	**Univariate analysis**		**Multivariate analysis**	
	**OR (95% CI)**	***p*-value**	**OR (95% CI)**	***p*-value**
Killip3/4^a^	37.56 (19.71–71.58)	<0.001	45.73 (12.03–173.78)	<0.001
Creatinine (100 μmol/L)^c^	1.39 (1.12–1.73)	0.002	1.33 (1.09–1.64)	0.006
Peak CK-MB(100 U/L)^d^	1.15 (1.07–1.23)	<0.001	1.30 (1.10–1.54)	0.002
Atrial fibrillation	6.77 (3.75–12.23)	<0.001	5.39 (1.45–20.04)	0.001
Age	1.05 (1.02–1.07)	<0.001	1.02 (0.96–1.08)	0.587
Diabetes	2.03 (1.17–3.55)	0.012	0.96 (0.29–3.20)	0.944
BNP (100 pg/ml)^b^	1.07 (1.05–1.10)	<0.001	0.99 (0.92–1.05)	0.692
LVEF (%)	0.90 (0.87–0.93)	<0.001	0.93 (0.86–1.00)	0.064
Lactate	1.33 (1.20–1.47)	<0.001	1.10 (0.99–1.21)	0.065
D–D dimer	1.19 (1.12–1.27)	<0.001	0.92 (0.77–1.09)	0.318
Post-PCI SBP (mmHg)	0.98 (0.97–0.99)	<0.001	1.02 (0.98– 1.06)	0.855
Post-PCI DBP (mmHg)	0.97 (0.95–0.98)	<0.001	0.97 (0.90–1.05)	0.213
Heart failure	5.11 (2.93–8.89)	<0.001	1.16 (0.28–4.85)	0.840
LM as culprit vessel	6.96 (2.94–16.46)	<0.001	9.78 (0.65–146.28)	0.098
Three-vessel disease	1.96 (1.15–3.36)	0.014	1.24 (0.38–3.98)	0.721
Post-PCI CTFC	1.02 (1.01–1.03)	0.011	0.97 (0.93–1.01)	0.109

a *Compared with Killip class 1/2*;

b *for every 100 pg/ml increase in BNP*;

c *for every 100 μmol/L increase in blood creatinine*;

d *for every 100 U/L increase in maximum CK-MB. BNP, brain natriuretic peptide; CK-MB, MB isoenzyme of creatine kianse; CTFC, corrected TIMI frame count; IHCA, in-hospital cardiac arrest; LM, left main artery; LVEF, left ventricular ejection fraction; NCA, non-cardiac arrest; DBP, dilation blood pressure; SBP, systolic blood pressure; PCI, percutaneous coronary intervention; PRCA, pre- revascularization cardiac arrest; POCA, post-revascularization cardiac arrest*.

### Risk Factors for All-Cause Mortality During Long-Term Follow-Up

Subsequently, we utilized univariate and multivariate COX regression analyses to assess predictors for long-term all-cause mortality. After adjustment for confounders, the following variables were identified as independent risk factors for long-term mortality (day 30 to end of study): POCA (HR 8.87, 95%CI 2.26–34.72, *p* = 0.002), older age (HR 1.04, 95%CI 1.01–1.07, *p* = 0.003), atrial fibrillation (HR 2.57, 95%CI 1.13–5.81, *p* = 0.024), heart failure (HR 2.78, 95%CI 1.61–4.81, *p* < 0.001), three-vessel coronary artery disease (HR 2.36, 95%CI 1.36–4.09 *p* = 0.002), and post-PCI CTFC (HR 0.98, 95%CI 0.96–1.00 *p* = 0.025) (Table 5).

**Table 5:** Predictors of all-cause mortality in long-term mortality (day 30 to end of study).

	**Univariate analysis**		**Multivariate analysis**	
	**HR (95% CI)**	***p*-value**	**HR (95% CI)**	***p*-value**
POCA	4.96 (2.19–11.22)	<0.001	8.87 (2.26–34.72)	0.002
Age	1.06 (1.05–1.07)	<0.001	1.04 (1.01–1.07)	0.003
Heart failure	3.56 (2.75–4.62)	<0.001	2.78 (1.61–4.81)	<0.001
Atrial fibrillation	3.74 (2.76–5.07)	<0.001	2.57 (1.13–5.81)	0.024
Three-vessel disease	2.48 (1.94–3.17)	<0.001	2.36 (1.36–4.09)	0.002
Post-PCI CTFC	1.01 (1–1.01)	0.043	0.98 (0.96–1.00)	0.025
STEMI^a^	1.43 (1.11–1.85)	0.006	1.55 (0.79–2.29)	0.203
Male gender	0.69 (0.53–0.88)	0.004	0.69 (0.40–0.1.18)	0.173
Killip3/4^b^	7.12 (5.52–9.18)	<0.001	1.66 (0.77–3.56)	0.196
LVEF (%)	0.93 (0.92–0.95)	<0.001	0.98 (0.94–1.01)	0.129
Post-PCI SBP (mmHg)	0.99 (0.98–0.99)	<0.001	1.00 (0.99–1.01)	0.994
Post-PCI DBP (mmHg)	0.97 (0.96–0.98)	<0.001	0.98 (0.96–1.01)	0.124
BNP (100 pg/ml)^c^	1.05 (1.04–1.07)	<0.001	0.98 (0.95–1.01)	0.124
Creatinine (100 μmol/L)^d^	1.21 (1.14–1.28)	<0.001	1.08 (0.79–1.48)	0.637
D–D dimer (mg/L)	1.11 (1.08–1.15)	<0.001	1.02 (0.94–1.10)	0.678
Maximum CK-MB (100 U/L)^e^	1.07 (1.03–1.10)	<0.001	1.03 (0.97–1.10)	0.337
Lactate(mmol/L)	1.07 (1.05–1.09)	<0.001	1.02 (0.97–1.78)	0.393
IABP	6.57 (4.74–9.10)	<0.001	1.52 (0.64–3.58)	0.341
Vasoactive drug	3.7 (2.74–5.00)	<0.001	0.86 (0.41–1.79)	0.686
Ventilation	6.41 (4.9–8.39)	<0.001	1.10 (0.52–2.32)	0.806
LM as culprit vessel	5.35 (3.34–8.56)	<0.001	2.16 (0.46–10.06)	0.326
Stent implantation	0.49 (0.36–0.66)	<0.001	0.72 (0.21–2.43)	0.595
Hypertension	1.38 (1.07–1.77)	0.013	1.04 (0.63–1.71)	0.869
Diabetes	1.65 (1.26–2.15)	<0.001	1.24 (0.72–2.13)	0.435

a *Compared with NSTEMI*;

b *compared with Killip class 1/2*;

c *For every 100 pg/ml increase in BNP*;

d *for every 100 μmol/L increase in blood creatinine*;

e *for every 100 U/L increase in maximum CK-MB. BNP, brain natriuretic peptide; CK-MB, MB isoenzyme of creatine kianse; CTFC, corrected TIMI frame count; IABP, intra-aortic balloon pump; LM, left main artery; LVEF, left ventricular ejection fraction; DBP, dilation blood pressure; SBP, systolic blood pressure; PCI, percutaneous coronary intervention; POCA, post-revascularization cardiac arrest; STEMI, ST-segment elevation myocardial infarction*.

## Discussion

### Main Findings

We investigated the incidence and prognostic influence of SCA (PRCA and POCA) in 1,956 consecutive patients with AMI who were admitted alive and underwent subsequently emergency PCI, and found that: (1) there were 175 patients (8.9%) and 57 patients (2.9%) experienced PRCA and POCA, respectively; (2) both PRCA and POCA were associated with higher 30-day mortality rate compared to the NCA group (8.0 and 70.2% vs. 2.9%, both *p* < 0.001); and (3) PRCA did not lead to poorer survival prognosis during long-term follow-up (median time of 917 days) in patients with AMI survived the first 30 days. In the contrast, POCA occurrence showed residual adverse influence on long-term survival prognosis in day 30 survivor population. (4) The independent predictors for POCA included Killip class 3/4, higher creatinine and maximum CK-MB, and atrial fibrillation. (5) Furthermore, multivariate analysis revealed that POCA was the strongest independent risk factor for mortality from day 30 to end of the study.

### Pre-Revascularization SCA Show Limited Adverse Impact on Long-Term Survival

Pre-hospital SCA has been demonstrated to be associated with poor survival prognosis in some studies. In contrast to patients with AMI in the thrombolysis era who started revascularization therapy by intravenous thrombolytic therapy since hospitalization, most patients in the present PCI era got recovery of coronary artery blood perfusion after percutaneous transluminal coronary angioplasty or stent implantation in the catheter laboratory. Most previous classified the patients who experienced SCA before artery recanalization in catheter laboratory as in-hospital cardiac arrest (IHCA) group and patients with SCA before arrival at hospital as out of hospital cardiac arrest (OHCA), which might lead to data biases in survival analysis. Indeed, our study observed pre-hospital SCA in 150 patients with myocardial infarction (7.7%) and 25 cases of SCA (1.3%) in the catheter laboratory before coronary artery recanalization. The mechanisms of pre-hospital SCA and pre-revascularization SCA in the emergency catheter laboratory should be similar, and the clinical characteristics are comparable.

Numerous studies have reported the adverse impact of pre-hospital SCA on patients with AMI. One report demonstrated that only a quarter of the OHCA victims got a return of spontaneous circulation, and only 1/10th of them could survive to discharge (8). Garot et al. (9) studied 186 consecutive patients with AMI complicated with successfully resuscitated OHCA who were subsequently admitted in cardiac care unit. They found a survival rate of 55.4% at the timing of discharge. However, no death event was described during 6-month follow-up, which might be due to the limited population number. In a large population study, Karam et al. enrolled 13,253 patients with STEMI managed by emergency medical system and reported 29.8% discharge mortality in OHCA compared to 4.0% in patients without SCA (10). Their study not only enrolled patients admitted alive but also those who died before arrival at hospital to ensure the reliability of mortality analysis. The discharge mortality rates of patients complicated with or without pre-hospital SCA were higher than that reported in the present study, which could be explained by different population inclusions. However, the study did not further investigate the post-discharge survival prognosis among patients who were discharged alive, which limited the prognosis predicting value on long-term outcome. Another study found a significantly higher in-hospital mortality rate in patients with STEMI complicated with OHCA than patients without SCA (13.8 vs. 3.4%), whereas the 1-year survival outcome in discharge survivors between the two groups was similar (11).

Sideris et al. (12) observed impaired admission survival (40.8%) and favorable 5-year survival after discharge (92.2%) among patients with pre-hospital SCA complicated with cardiogenic origin (ACS) (12). Similarly, Kvakkestad et al. (11) also reported a high 8-year survival (77.6%) among patients with AMI with witnessed OHCA, who survived the first 30 days. Previous studies suggested that the 5-year survival rates after discharge in OHCA populations varied from 41 to 84% depending on different etiologies and inclusion criteria (13–15). However, the good long-term survival outcome of patients with PRCA of presumed cardiac origin, who survived the first 30 days, is convincing.

The main etiology underlying PRCA occurrence seems to be severe ventricular arrythmia. Demirel et al. (16) analyzed the 4,653 patients with STEMI and found that 326 patients (7.0%) experienced pre-hospital cardiac arrest due to pVT or VF, which was similar with the rate of PRCA (8.9%, 175/1,956) recorded in our study. In published reports, VF/pVT contributed to nearly 80% of pre-hospital SCA among patients with AMI (10, 17).

The pathophysiology of VF/pVT during AMI is associated with a complex interaction of both environmental and genetic causes, including acute myocardial ischemia, ongoing ischemia, electrolyte disorder, and macro-reentry between ischemic and non-ischemic ventricular walls (18, 19). Although one would expect extensive ischemia regions and prolonged ischemia in AMI victims with witnessed SCA, recent evidence suggested that rhythmic vulnerability might partially account for some SCA cases, in which patients with AMI exhibit susceptibility to develop shockable arrhythmias soon after acute myocardial ischemia onset (10). Differed from patients with POCA, we did not find a significant difference in the PRCA group when compared to the NCA group upon proportion of three-vessel disease, stent implantation, and heart failure admission events. We hypothesis that alterations on the resting membrane potential due to acute obstruction of coronary flow play a vital role in the onset of VF/pVT rhythm. And most cardiac functions of patients with AMI will be reserved once the restoration of coronary flow is achieved by immediate PCI therapy. This may partially explain the outstanding long-term survival outcome in patients with PRCA, who survived the first 30 days.

### Post-Revascularization SCA Show Adverse Prognostic Influence Beyond the First 30 Days

In-hospital cardiac arrest was associated with high mortality at discharge, which varies from 47 to 85% depending on different criteria and inclusions (20–22). This contemporary study demonstrated a high 30-day mortality of 70.2% in patients with AMI with POCA, and a high cumulative mortality of 36.3% from day 30 to end of the study (median follow-up time 917 days). Chen et al. (23) reported a 1-year survival rate of 60.2% in White race and 43.6% in Black race among IHCA survivors, which were comparable with our results based on Asian people (23). It has been reported that early IHCA, which contain a proportion of PRCA according to definition, were associated with relatively good survival when compared to delayed IHCA among STEMI population receiving primary PCI (20). The timing of SCA in the acute coronary syndrome population implies a significant determinant of clinical outcomes and underlying cause a difference in the etiologies. Differed from patients with OHCA, who often occur unexpectedly and most are of cardiac etiology, IHCA often deteriorate cardiac function previously and develop cardiogenic shock before terminal cardiac arrest (24).

Quite different from early SCA events, which are due to more easily reversible cause like shockable rhythm (pVT or VF), late SCA events often mean a multi-organ failure diseases and refractory shock. The POCA group appeared to be sicker even on admission than the PRCA group, with more than twice the lactate, higher BNP concentration, more proportion of patients with Killip class 3/4, more often IABP and ventilation use, and nearly 7 years older age on average. Additionally, coronary angiography demonstrated a low proportion of complete blood flow restoration, more frequent three-vessel disease, and more often LM or LAD ostial stenosis in patients with POCA than with NCA. Current research works focused on the antiarrhythmic effects of ischemic pre- and postconditioning. Andreas et al. found that preconditioning exerted antiarrhythmic properties during ischemia, whereas postconditioning exhibited antiarrhythmic effects after reperfusion, through downregulating ischemia-related miRNAs (25). Theofilos et al. observed weaker antiarrhythmic effects of postconditioning than preconditioning (26), indicating the antiarrhythmic properties of postconditioning remain to be further explored.

We found increased creatinine level is an independent risk factor for POCA, indicating a vital role of impaired renal function in the occurrence of fatal arrhythmia. Ventricular arrhythmia and sudden cardiac death risk are increased in patients with renal failure (27). Hyperkalemia, hypocalcemia, and hypomagnesemia have been demonstrated to be common complications of renal insufficiency. They show a cumulative effect on the atrioventricular and intraventricular conduction and facilitate ventricular fibrillation (28). And issues, such as serum phosphates and iron, parathyroid hormone level, renal function, hemoglobin and hematocrit, pH, inflammatory markers, and standard 12-lead ECG, were also suggested to be monitored during treatment for electrolyte disorders (29). Our present study observed higher BNP levels in patients with PRCA and POCA. BNP has been proved to have protective effects against collagen formation and accumulation, and pathologic cardiac remodeling, which may *via* inhibition of rennin–angiotensin system, and systemic and renal sympathetic activity. BNP relatively increases heart rate and sinoatrial node activity in basal conditions (30). Moreover, BNP was demonstrated to increase cardiac conduction velocity, *via* activating multiple natriuretic peptide receptors, independent of cycle length (31). However, multivariate analysis showed higher BNP neither a risk factor for POCA occurrence nor predictor for adverse long-term survival outcome. GUIDE-IT trial (32) and PRIMA II (33) failed to observe a favorable effect of BNP-guided treatment on all-cause mortality, heart failure readmissions, and median days alive outside of the hospital. However, some studies observed a slight favorable effect of BNP-guided therapy among patients younger than 72 years of age (34, 35), suggesting the significance of monitoring BNP may differ between different populations.

### Independent Predictors Associated With Worse Survival Prognosis

Unsurprisingly, older age and heart failure, which indicated weak body condition, were identified to be risk factors for long-term mortality. Despite similar degree of culprit vessel stenosis and pre-PCI TIMI flow grade, there was a lower rate of TIMI 3 flow achievement after intervention in the POCA group (86.0%), when compared with NCA (93.4%) group. The higher proportion of three-vessel coronary disease and high CTFC in patients with POCA also indicted difficulties in obtaining completed recovery of coronary reperfusion. Our results advocate the conclusion that early restoration of complete reperfusion (TIMI 3 flow) of the infarct-related artery in patients with AMI offers a mortality benefit (36, 37). We observed a relatively better 30-day survival prognosis in POCA with shockable rhythm, compared to POCA with non-shockable rhythm, which was consist with the findings of previous reports (22, 38). Similarly, non-shockable rhythm was more frequently noted in initial arrhythmia of POCA (23). Unlike shockable rhythm which could be rapidly reversed by electric defibrillation, non-shockable rhythm is associated with multi-organ failure and unfavorable body condition. We demonstrated atrial fibrillation to be a strong independent predictor for long-term mortality in patients with SCA with AMI. In line with some studies, we found a significantly higher proportion of new-onset atrial fibrillation in patients with AMI complicated with SCA (5.1% in PRCA and 19.3% in POCA, respectively), compared to patients without SCA (2.1%) (39). In a systemic meta-analysis, new-onset atrial fibrillation was identified to be associated with poor survival prognosis in AMI population (40). Despite studies showed conflicting evidence in determining the influence of new-onset atrial fibrillation on 30-day mortality, the adverse influence of both previous and new-onset atrial fibrillation after AMI on long-term survival prognosis was convincing (41).

### Limitations

There are several limitations to be considered in our study. First, patients with AMI who died between symptom onset and arrival at the hospital were not included in the cohort study. Second, some out-of-hospital resuscitation-related variables were unavailable, including duration of total arrest time, cardiopulmonary resuscitation time, and initial arrhythmia at SCA. Third, we failed to collect data of Glasgow coma scale score to evaluate neurological outcomes. Additionally, troponin I and troponin T were not involved. Our department changed troponin assay kits during study period, and the early troponin assay kits only had a maximum of 50 μg/L, resulting in a bias in statistical analysis. Fourth, we chose atrial fibrillation rhythm as ECG predictor for SCA. However, other outcome-related ECG issues, like interatrial block, deep terminal negativity of the P wave in V1, prolonged QT intervals, QRS duration, bundle branch block, ST segment depression, and premature ventricular contractions, were not considered for data missing (42).

## Conclusions

Post-revascularization cardiac arrest was associated with a higher 30-day and long-term mortality rate in patients with AMI who underwent emergency PCI. By contrast, PRCA did not show a residual adverse effect on survival rate beyond day 30. Unfavorable prognostic factors for long-term survival included POCA, older age, atrial fibrillation, heart failure, three-vessel disease, and high CTFC. Our study indicated different survival outcomes of patients with SCA according to SCA timing, and strict medication guidance and more frequent clinical follow-up are suggested among POCA population.

## Data Availability Statement

The raw data supporting the conclusions of this article will be made available by the authors, without undue reservation.

## Ethics Statement

The studies involving human participants were reviewed and approved by Ethics Committee in clinical research of the First Affiliated Hospital of Wenzhou Medical University. The patients/participants provided their written informed consent to participate in this study.

## Author Contributions

All authors listed have made a substantial, direct and intellectual contribution to the work, and approved it for publication.

## Funding

This study was supported by the National Natural Science Foundation of China (81600341), the Natural Science Foundation of Zhejiang Province (LQ15H020005), and Wenzhou Science Technology Bureau Foundation (Y20190616).

## Conflict of Interest

The authors declare that the research was conducted in the absence of any commercial or financial relationships that could be construed as a potential conflict of interest.

## Publisher's Note

All claims expressed in this article are solely those of the authors and do not necessarily represent those of their affiliated organizations, or those of the publisher, the editors and the reviewers. Any product that may be evaluated in this article, or claim that may be made by its manufacturer, is not guaranteed or endorsed by the publisher.

## References

[1] Spaulding CM Joly LM Rosenberg A Monchi M Weber SN Dhainaut JF. Immediate coronary angiography in survivors of out-of-hospital cardiac arrest. N Engl J Med. (1997) 336:1629–33. 10.1056/NEJM199706053362302 9171064

[2] Zeyons F Jesel L Morel O Kremer H Messas N Hess S. Out-of-hospital cardiac arrest survivors sent for emergency angiography: a clinical score for predicting acute myocardial infarction. Eur Heart J Acute Cardiovasc Care. (2017) 6:103–11. 10.1177/2048872616683525 28304194

[3] O'Gara PT Kushner FG Ascheim DD Casey DE Jr. Chung MK de Lemos JA. 2013 ACCF/AHA guideline for the management of ST-elevation myocardial infarction: executive summary: a report of the American College of Cardiology Foundation/American Heart Association Task Force on Practice Guidelines. Circulation. (2013) 127:529–55. 10.1161/CIR.0b013e3182742c84 23247303

[4] Peberdy MA Callaway CW Neumar RW Geocadin RG Zimmerman JL Donnino M. Part 9: post-cardiac arrest care: 2010 American Heart Association Guidelines for Cardiopulmonary Resuscitation and Emergency Cardiovascular Care. Circulation. (2010) 122:S768–86. 10.1161/CIRCULATIONAHA.110.971002 20956225

[5] Strote JA Maynard C Olsufka M Nichol G Copass MK Cobb LA. Comparison of role of early (less than six hours) to later (more than six hours) or no cardiac catheterization after resuscitation from out-of-hospital cardiac arrest. Am J Cardiol. (2012) 109:451–4. 10.1016/j.amjcard.2011.09.036 22100026 PMC3270205

[6] Zanuttini D Armellini I Nucifora G Carchietti E Trillo G Spedicato L. Impact of emergency coronary angiography on in-hospital outcome of unconscious survivors after out-of-hospital cardiac arrest. Am J Cardiol. (2012) 110:1723–8. 10.1016/j.amjcard.2012.08.006 22975468

[7] Kim MJ Ro YS Shin SD Song KJ Ahn KO Hong SO. Association of emergent and elective percutaneous coronary intervention with neurological outcome and survival after out-of-hospital cardiac arrest in patients with and without a history of heart disease. Resuscitation. (2015) 97:115–21. 10.1016/j.resuscitation.2015.08.019 26384459

[8] Mozaffarian D Benjamin EJ Go AS Arnett DK Blaha MJ Cushman M. Heart disease and stroke statistics−2015 update: a report from the American Heart Association. Circulation. (2015) 131:e29–322. 10.1161/CIR.0000000000000152 25520374

[9] Garot P Lefevre T Eltchaninoff H Morice MC Tamion F Abry B. Six-month outcome of emergency percutaneous coronary intervention in resuscitated patients after cardiac arrest complicating ST-elevation myocardial infarction. Circulation. (2007) 115:1354–62. 10.1161/CIRCULATIONAHA.106.657619 17353440

[10] Karam N Bataille S Marijon E Tafflet M Benamer H Caussin C. Incidence, Mortality, and Outcome-Predictors of Sudden Cardiac Arrest Complicating Myocardial Infarction Prior to Hospital Admission. Circ Cardiovasc Interv. (2019) 12:e007081. 10.1161/CIRCINTERVENTIONS.118.007081 30608874

[11] Kvakkestad KM Sandvik L Andersen GO Sunde K Halvorsen S. Long-term survival in patients with acute myocardial infarction and out-of-hospital cardiac arrest: a prospective cohort study. Resuscitation. (2018) 122:41–7. 10.1016/j.resuscitation.2017.11.047 29155294

[12] Sideris G Voicu S Yannopoulos D Dillinger J-G Adjedj J Deye N. Favourable 5-year postdischarge survival of comatose patients resuscitated from out-of-hospital cardiac arrest, managed with immediate coronary angiogram on admission. Eur Heart J Acute Cardiovasc Care. (2014) 3:183–91. 10.1177/2048872614523348 24569450

[13] Geri G Dumas F Bougouin W Varenne O Daviaud F Pène F. Immediate percutaneous coronary intervention is associated with improved short- and long-term survival after out-of-hospital cardiac arrest. Circ Cardiovasc Intervent. (2015) 8:e002303. 10.1161/CIRCINTERVENTIONS.114.002303 26453685

[14] Bergman R Hiemstra B Nieuwland W Lipsic E Absalom A van der Naalt J. Long-term outcome of patients after out-of-hospital cardiac arrest in relation to treatment: a single-centre study. Eur Heart J Acute Cardiovasc Care. (2016) 5:328–38. 10.1177/2048872615590144 26068962

[15] Dumas F White L Stubbs BA Cariou A Rea TD. Long-term prognosis following resuscitation from out of hospital cardiac arrest: role of percutaneous coronary intervention and therapeutic hypothermia. J Am College Cardiol. (2012) 60:21–7. 10.1016/j.jacc.2012.03.036 22742398

[16] Demirel F Rasoul S Elvan A Ottervanger JP Dambrink JH Gosselink AT. Impact of out-of-hospital cardiac arrest due to ventricular fibrillation in patients with ST-elevation myocardial infarction admitted for primary percutaneous coronary intervention: impact of ventricular fibrillation in STEMI patients. Eur Heart J Acute Cardiovasc Care. (2015) 4:16–23. 10.1177/2048872614547448 25114328

[17] Zeliaś A Stepińska J Andres J Trabka-Zawicki A Sadowski J Zmudka K. Ten-year experience of an invasive cardiology centre with out-of-hospital cardiac arrest patients admitted for urgent coronary angiography. Kardiol Polska. (2014) 72:687–99. 10.5603/KP.a2014.0088 24846357

[18] Gheeraert PJ Henriques JP De Buyzere ML Voet J Calle P Taeymans Y. Out-of-hospital ventricular fibrillation in patients with acute myocardial infarction: coronary angiographic determinants. J Am College Cardiol. (2000) 35:144–50. 10.1016/S0735-1097(99)00490-8 10636272

[19] Tang L Deng C Long M Tang A Wu S Dong Y. Thrombin receptor and ventricular arrhythmias after acute myocardial infarction. Mol Med. (2008) 14:131–40. 10.2119/2007-00097.Tang 18224254 PMC2213895

[20] Vallabhajosyula S Vallabhajosyula S Bell MR Prasad A Singh M White RD. Early vs. delayed in-hospital cardiac arrest complicating ST-elevation myocardial infarction receiving primary percutaneous coronary intervention. Resuscitation. (2019) 148:242–50. 10.1016/j.resuscitation.2019.11.007 31759071

[21] Kolte D Khera S Aronow WS Palaniswamy C Mujib M Ahn C. Regional variation in the incidence and outcomes of in-hospital cardiac arrest in the United States. Circulation. (2015) 131:1415–25. 10.1161/CIRCULATIONAHA.114.014542 25792560

[22] Girotra S Nallamothu BK Spertus JA Li Y Krumholz HM Chan PS. Trends in survival after in-hospital cardiac arrest. N Engl J Med. (2012) 367:1912–20. 10.1056/NEJMoa1109148 23150959 PMC3517894

[23] Chen LM Nallamothu BK Spertus JA Tang Y Chan PS. Racial differences in long-term outcomes among older survivors of in-hospital cardiac arrest. Circulation. (2018) 138:1643–50. 10.1161/CIRCULATIONAHA.117.033211 29987159

[24] Morrison LJ Neumar RW Zimmerman JL Link MS Newby LK McMullan PW. Strategies for improving survival after in-hospital cardiac arrest in the United States: 2013 consensus recommendations: a consensus statement from the American Heart Association. Circulation. (2013) 127:1538–63. 10.1161/CIR.0b013e31828b2770 23479672

[25] Spannbauer A Traxler D Lukovic D Zlabinger K Winkler J Gugerell A. Effect of ischemic preconditioning and postconditioning on exosome-rich fraction microRNA levels, in relation with electrophysiological parameters and ventricular arrhythmia in experimental closed-chest reperfused myocardial infarction. Int J Mol Sci. (2019) 20:2140. 10.3390/ijms20092140 31052231 PMC6540096

[26] Kolettis TM Vilaeti AD Tsalikakis DG Zoga A Valenti M Tzallas AT. Effects of pre- and postconditioning on arrhythmogenesis in the *in vivo* rat model. J Cardiovasc Pharmacol Ther. (2013) 18:376–85. 10.1177/1074248413482183 23524840

[27] Al-Khatib SM Stevenson WG Ackerman MJ Bryant WJ Callans DJ Curtis AB. 2017 AHA/ACC/HRS guideline for management of patients with ventricular arrhythmias and the prevention of sudden cardiac death: a report of the American College of Cardiology/American Heart Association Task Force on Clinical Practice Guidelines and the Heart Rhythm Society. J Am Coll Cardiol. (2018) 72:e91–220. 10.1016/j.jacc.2017.10.054 29097296

[28] Bonato FOB Canziani MEF. Ventricular arrhythmia in chronic kidney disease patients. J Bras Nefrol. (2017) 39:186–95. 10.5935/0101-2800.20170033 29069243

[29] Mozos I. Laboratory markers of ventricular arrhythmia risk in renal failure. Biomed Res Int. (2014) 2014:509204. 10.1155/2014/509204 24982887 PMC4058221

[30] Caprnda M Zulli A Shiwani HA Kubatka P Filipova S Valentova V. The therapeutic effect of B-type natriuretic peptides in acute decompensated heart failure. Clin Exp Pharmacol Physiol. (2020) 47:1120–33. 10.1111/1440-1681.13290 32083749

[31] Azer J Hua R Krishnaswamy PS Rose RA. Effects of natriuretic peptides on electrical conduction in the sinoatrial node and atrial myocardium of the heart. J Physiol. (2014) 592:1025–45. 10.1113/jphysiol.2013.265405 24344164 PMC3948561

[32] Felker GM Anstrom KJ Adams KF Ezekowitz JA Fiuzat M Houston-Miller N. Effect of natriuretic peptide-guided therapy on hospitalization or cardiovascular mortality in high-risk patients with heart failure and reduced ejection fraction: a randomized clinical trial. JAMA. (2017) 318:713–20. 10.1001/jama.2017.10565 28829876 PMC5605776

[33] Stienen S Salah K Moons AH Bakx AL van Pol P Kortz RAM. NT-proBNP (N-Terminal pro-B-Type Natriureticcx Peptide)-guided therapy in acute decompensated heart failure: PRIMA II randomized controlled trial (Can NT-ProBNP-Guided therapy during hospital admission for acute decompensated heart failure reduce mortality and readmissions?). Circulation. (2018) 137:1671–83. 10.1161/CIRCULATIONAHA.117.029882 29242350

[34] Khan MS Siddiqi TJ Usman MS Sreenivasan J Fugar S Riaz H. Does natriuretic peptide monitoring improve outcomes in heart failure patients? A systematic review and meta-analysis. Int J Cardiol. (2018) 263:80–7. 10.1016/j.ijcard.2018.04.049 29685696

[35] Pufulete M Maishman R Dabner L Mohiuddin S Hollingworth W Rogers CA. Effectiveness and cost-effectiveness of serum B-type natriuretic peptide testing and monitoring in patients with heart failure in primary and secondary care: an evidence synthesis, cohort study and cost-effectiveness model. Health Technol Assess. (2017) 21:1–150. 10.3310/hta21400 28774374 PMC5554865

[36] Kim DW Her SH Park MW Cho JS Kim TS Kang H. Impact of post-procedural TIMI flow on long-term clinical outcomes in patients with acute myocardial infarction. Int Heart J. (2017) 58:674–85. 10.1536/ihj.16-448 28966314

[37] Vogt A von Essen R Tebbe U Feuerer W Appel KF Neuhaus KL. Impact of early perfusion status of the infarct-related artery on short-term mortality after thrombolysis for acute myocardial infarction: retrospective analysis of four German multicenter studies. J Am Coll Cardiol. (1993) 21:1391–5. 10.1016/0735-1097(93)90314-Q 8473646

[38] Ohlsson MA Kennedy LM Juhlin T Melander O. Evaluation of pre-arrest morbidity score and prognosis after resuscitation score and other clinical variables associated with in-hospital cardiac arrest in southern Sweden. Resuscitation. (2014) 85:1370–4. 10.1016/j.resuscitation.2014.07.009 25079198

[39] Lee KH Jeong MH YoungkeunAhn Kim SS Rhew SH Jeong YW. One-year clinical impact of cardiac arrest in patients with first onset acute ST-segment elevation myocardial infarction. Int J Cardiol. (2014) 175:147–53. 10.1016/j.ijcard.2014.05.002 24856807

[40] Ren Y Zeng RX Li JJ Guo LH He DY Li Y. Relation of C-reactive protein and new-onset atrial fibrillation in patients with acute myocardial infarction: a systematic review and meta-analysis. Int J Cardiol. (2015) 190:268–70. 10.1016/j.ijcard.2015.04.152 25932802

[41] Gorenek B Kudaiberdieva G. Atrial fibrillation in acute ST-elevation myocardial infarction: clinical and prognostic features. Curr Cardiol Rev. (2012) 8:281–9. 10.2174/157340312803760857 22920476 PMC3492812

[42] Mozos I Caraba A. Electrocardiographic predictors of cardiovascular mortality. Dis Markers. (2015) 2015:727401. 10.1155/2015/727401 26257460 PMC4519551

